# Calcium Promotes T6SS-Mediated Killing and Aggregation between Competing Symbionts

**DOI:** 10.1128/spectrum.01397-22

**Published:** 2022-12-01

**Authors:** Lauren Speare, Aundre Jackson, Alecia N. Septer

**Affiliations:** a Department of Earth, Marine & Environmental Sciences, University of North Carolina, Chapel Hill, North Carolina, USA; b Department of Microbiology, Oregon State University, Corvallis, Oregon, USA; Centre national de la recherche scientifique, Aix-Marseille Université

**Keywords:** type VI secretion system, calcium, regulation, competition, *Aliivibrio*

## Abstract

Bacteria use a variety of strategies to exclude competitors from accessing resources, including space within a host niche. Because these mechanisms are typically costly to deploy, they are often tightly regulated for use in environments where the benefits outweigh the energetic cost. The type VI secretion system (T6SS) is a competitive mechanism that allows inhibitors to kill competing microbes by physically puncturing and translocating cytotoxic effectors directly into neighboring competitor cells. Although T6SSs are encoded in both symbiotic and free-living taxa where they may be actively secreting into the extracellular milieu during growth in liquid culture, there is little evidence for bacteria engaging in T6SS-mediated, contact-dependent killing under low-viscosity liquid conditions. Here, we determined that calcium acts as a pH-dependent cue to activate the assembly of an antibacterial T6SS in a Vibrio fischeri light organ symbiont in a low-viscosity liquid medium. Moreover, competing V. fischeri isolates formed mixed-strain aggregates that promoted the contact necessary for T6SS-dependent elimination of a target population. Our findings expand our knowledge of V. fischeri T6SS ecology and identify a low-viscosity liquid condition where cells engage in contact-dependent killing.

**IMPORTANCE** Microbes deploy competitive mechanisms to gain access to resources such as nutrients or space within an ecological niche. Identifying when and where these strategies are employed can be challenging given the complexity and variability of most natural systems; therefore, studies evaluating specific cues that conditionally regulate interbacterial competition can inform the ecological context for such competition. In this work, we identified a pH-dependent chemical cue in seawater, calcium, which promotes activation of a contact-dependent interbacterial weapon in the marine symbiont Vibrio fischeri. This finding underscores the importance of using ecologically relevant salts in growth media and the ability of bacterial cells to sense and integrate multiple environmental cues to assess the need for a weapon. Identification of these cues provides insight into the types of environments where employing a weapon is advantageous to the survival and propagation of a bacterial population.

## OBSERVATION

Molecular weapons allow microbes to eliminate competitors of an ecological niche. Symbiotic microbes often regulate competitive mechanisms in response to environmental cues, limiting use of these energetically expensive weapons to conditions where the benefits outweigh the costs (1, 2). The type VI secretion system (T6SS) is a widely distributed interbacterial weapon that can provide a competitive advantage during symbiotic initiation (3–6). T6SSs are large, proteinaceous structures that act like molecular weapons to deliver effector proteins directly into competing cells (7). T6SSs require direct contact between competing cells types to deliver cytotoxic effectors, although contact-independent killing has been recently described for Yersinia pseudotuberculosis (8). While some taxa exhibit active T6SS protein secretion of a key T6SS structural protein, hemolysin-coregulated protein (Hcp), in liquid media (9–11), contact-dependent killing in such conditions has not been described, presumably due to the lack of cell-cell contact. Here, we identify a new liquid condition that promotes both T6SS activity and cell-cell contact using the marine bacterium Vibrio fischeri.

V. fischeri encodes a strain-specific T6SS on chromosome II (T6SS2) that eliminates competitors *in vitro* and during symbiosis establishment with Euprymna scolopes squid (3, 12–14). Juvenile squid hatch with an aposymbiotic light organ that is quickly populated by free-living V. fischeri. Although not necessary for symbiosis establishment in the absence of a competitor, strains that encode T6SS2 have a competitive advantage over strains lacking T6SS2: T6SS2^+^ strains can eliminate T6SS^−^ strains in cocolonized crypts, resulting in clonally colonized crypts where the incompatible strain types are spatially separated in different crypt spaces within the host (3, 13). T6SS2-mediated killing requires two host-specific cues: (i) high-viscosity liquids and surfaces activate T6SS2 expression and sheath assembly (15), and (ii) a combination of high-viscosity and neutral-to-acidic pH (7.5 and 6.5) promotes cell-cell contact (15, 16). However, low-viscosity liquid conditions that promote T6SS2 activity have not yet been identified. Previous work revealed that calcium induces cellulose-dependent aggregation in low-viscosity liquid (17, 18); therefore, we wondered whether T6SS2 is also active under these conditions, where cells may come into contact with competitors.

**FIG 1:**
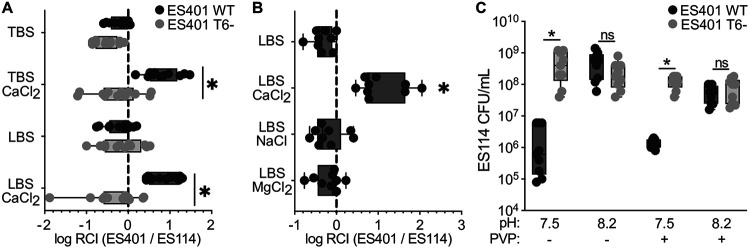
Calcium activates T6SS2-mediated killing in liquid media. Results of coincubation assays in TBS or LBS liquid with or without 10 mM CaCl_2_ (A), LBS liquid with or without 10 mM CaCl_2_, 10 mM NaCl, or 10 mM MgCl_2_ (B), or LBS liquid plus 10 mM CaCl_2_ with or without 5% polyvinylpyrrolidone (PVP) at pH 7.5 or 8.2 (C). Experiments were performed with strains ES114 and ES401 wild type (WT; dark gray) or T6SS2 mutant (T6^−^; light gray). Results were calculated from CFUs and are displayed as log relative competitive index (RCI) values (A and B) or ES114 CFU per milliliter at 15 h (C). Log RCI values were calculated from the ES401:ES114 ratio of CFUs collected at 12 h (A) or 15 h (B) after incubation, divided by the ratio of these strains at the beginning of the experiment. Asterisks indicate significantly different values (Sidak’s multiple-comparison test, *P < *0.0001) between coincubations with ES401 WT versus T6^−^ in a given medium (A and C) or between different conditions (B). Experiments were performed three times, and combined data are shown (A, *n* = 12; B and C, *n* = 9).

To determine whether calcium impacts T6SS2 activity in liquid, we performed coincubation assays using two incompatible V. fischeri strains: a target strain, ES114, that does not encode T6SS2, and an inhibitor strain, ES401, that kills ES114 using T6SS2 (15, 19, 20). Strains were differentially tagged with unique antibiotic resistance genes, mixed in a 1:1 ratio, and incubated for 12 to 15 h with shaking in low-viscosity (centipoise [cP] of 1) liquid Luria-Bertani with salts (LBS) or tryptone broth-saline (TBS) (18) media without or with 10 mM CaCl_2_, which is comparable to the calcium concentration in seawater (21). For each coincubation assay, CFUs were collected for each strain at the beginning and end of the experiment and used to calculate the log relative competitive index (RCI) values to evaluate whether ES401 outcompeted ES114. Log RCI values were significantly greater than zero in LBS and TBS with CaCl_2_, yet not in media without CaCl_2_ (Fig. 1A), suggesting that ES401 outcompeted ES114 in the presence of calcium chloride. When we repeated these experiments with an ES401 T6SS2 mutant, which had a disruption in an essential T6SS2 structural gene (*tssF_2/vasA_2*), log RCI values were not significantly greater than zero under any condition and were significantly different from those for the wild-type coincubations (Fig. 1A), suggesting that ES401 used T6SS2 to outcompete ES114 in liquid with calcium chloride. To determine whether calcium, chloride, or divalent cations generally activate T6SS2 in liquid, we repeated these experiments in LBS liquid supplemented with 10 mM CaCl_2_, NaCl, or MgCl_2_. Log RCI values were not significantly greater than zero for NaCl and MgCl_2_ treatments and were significantly lower than the CaCl_2_ treatment (Fig. 1B), suggesting that calcium specifically promotes T6SS2 killing in liquid.

We previously showed that pH controls T6SS killing by ES401 in high-viscosity liquid (16); therefore, we predicted that pH may similarly affect T6SS activity in low-viscosity liquid supplemented with calcium. To test our prediction, we coincubated ES114 with ES401 wild type or a T6SS2 mutant (*tssF_2^−^*) in low-viscosity liquid LBS (1 cP) media supplemented with 10 mM CaCl_2_ that was buffered to pH 7.5 or 8.2. The ES114 CFUs recovered at the end of coincubations were significantly greater with the T6SS2 mutant than in coincubations with the wild type at pH 7.5, yet they were not significantly different between ES401 strains at pH 8.2 (Fig. 1C). We observed the same phenotype when we repeated this experiment in high-viscosity liquid (5% polyvinylpyrrolidone [PVP], cP of 152) (15) medium supplemented with calcium (Fig. 1C), suggesting that T6SS-dependent killing in both calcium and high-viscosity media is pH dependent.

**FIG 2:**
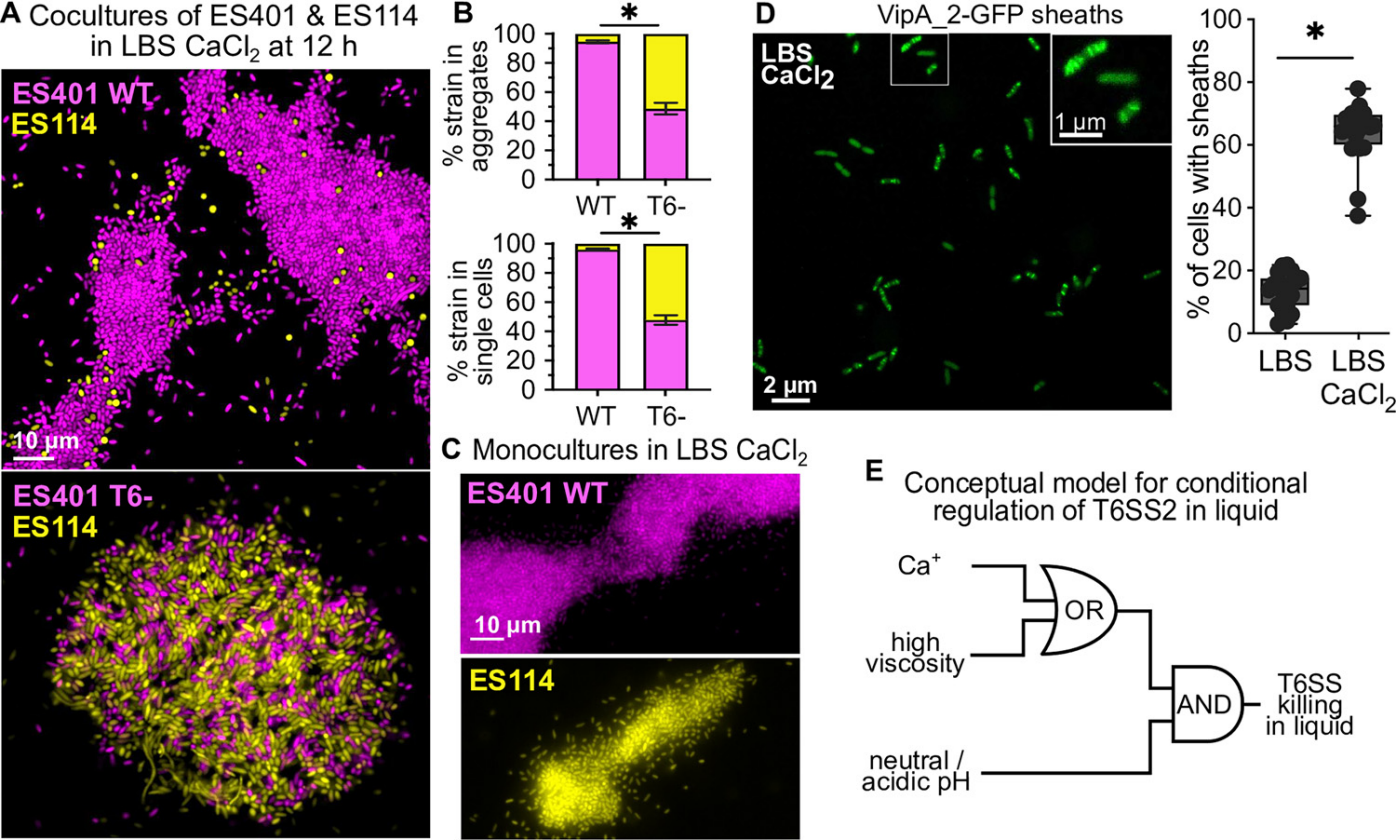
Calcium promotes cell-cell contact and sheath assembly in liquid media. (A and C) Representative fluorescence microscopy images of cocultures of ES401 WT or T6^−^ (magenta) incubated with ES114 (yellow) (A) or monocultures of each strain in LBS plus 10 mM CaCl_2_ for 12 h (C). Each experiment was performed three times with one biological replicate and four fields of view (*n* = 12). (B) Results from mixed-strain coaggregation assays, displayed as the percentage of each strain (ES114, yellow; ES401, magenta) within aggregates (top) or in the single-cell fraction (bottom). (D) Representative GFP-labeled images of ES401 harboring a VipA_2-GFP expression vector incubated in LBS with 10 mM CaCl_2_ and supplemented with 0.5 mM isopropyl-β-d-thiogalactopyranoside (IPTG) for 3 h. Percentages are shown for VipA_2-GFP-expressing cells that contained at least one sheath after being incubated in LBS with or without CaCl_2_ and supplemented with IPTG for 3 h. The asterisk indicates a significantly different percentage of cells with sheaths between media types (Student's *t* test, *P < *0.0001). Each experiment was performed twice with two biological replicates and five fields of view (*n* = 20). (E) Conceptual model for the conditional regulation of the V. fischeri T6SS2 in liquid media displayed as a logic gate. The V. fischeri T6SS2 facilitates interbacterial killing both on surfaces and in liquid environments when specific conditions are met. Either calcium or high viscosity combined with neutral or acidic pH promote T6SS activity in a liquid environment.

Given that T6SS2-mediated killing requires cell-cell contact (3, 13), we predicted that calcium promotes both cell-cell contact and T6SS2 expression in liquid media. We used fluorescence microscopy to test our prediction that calcium promotes cell-cell contact and T6SS expression. To determine whether the competing strains made contact in coculture, we visualized differentially tagged ES114 and ES401 strains grown in LBS liquid with 10 mM CaCl_2_ for 12 h. In LBS liquid without calcium, cells were physically dispersed (15); however, with the addition of calcium we observed large aggregates (Fig. 2A). In cocultures with wild-type ES401, 95% of the aggregated cells were ES401 (Fig. 2B) and the majority of ES114 cells present were rounded (Fig. 2A), suggesting they were intoxicated with at least one T6SS effector. In cocultures with the ES401 *tssF_2* mutant, aggregates contained a relatively equal proportion of ES401 and ES114, 49% and 51%, respectively (Fig. 2A and B). Aggregates were also observed in monocultures of ES401 (T6SS2^+^) and ES114 (T6SS2^−^) in LBS liquid plus 10 mM CaCl_2_ (Fig. 2C), suggesting that calcium promotes aggregation in liquid independently of T6SS2.

Previous work demonstrated that visualizing the percentage of cells with T6SS2 sheaths is a good indicator of T6SS activation in V. fischeri, because a T6SS2-specific transcriptional reporter and protein expression are low in LBS liquid, where few sheaths are observed (15). VipA is a subunit of the T6SS sheath and by tagging it with a green fluorescent protein (GFP) molecule, we could visualize sheath assembly in live cells (Fig. 2D). We visualized T6SS2-GFP sheaths by incubating strain ES401 harboring a VipA_2-GFP expression vector (3) in liquid LBS with or without 10 mM CaCl_2_ for 3 h. A significantly higher percentage of cells contained sheaths in the presence of calcium (64%) compared to sheaths in cultures without calcium (14%) (Fig. 2D), indicating the T6SS2 is active in cells grown in the presence of calcium. Notably, aggregates were not observed in these experiments, suggesting T6SS is activated directly in response to calcium rather than via cell-cell contact. Taken together, these data suggest that calcium acts as a cue to promote T6SS function by activating T6SS sheath assembly and mixed-strain aggregation in liquid media. Thus, our findings provide evidence of contact-dependent T6SS killing in a low-viscosity liquid condition in response to an ecologically relevant cue.

Regulation of T6SSs by environmental conditions has been well established in a number of free-living and symbiotic taxa. Although calcium and other divalent cations can repress T6SS expression and/or activity of Salmonella enterica (22) and Pseudomonas aeruginosa PAO1 (23), our findings suggest calcium can also be an ecologically relevant activator of T6SS. Although our data suggest that V. fischeri may engage in T6SS killing in calcium-containing low-viscosity liquid environments outside of the *E. scolopes* light organ, the pH of seawater (8.2) is not permissive to T6SS-dependent competition. However, it is possible that T6SS2 may be active in marine microhabitats with lower pH (24) and therefore provide V. fischeri with a competitive advantage in those niches.

Although we did not identify how calcium promotes cell-cell contact in liquid, several possible mechanisms could explain this observation. First, the contact may be facilitated by TasL, a newly described putative lipoprotein encoded in the V. fischeri T6SS2 gene cluster (13). TasL promotes inhibitor-target contact in high-viscosity liquid by forming large, mixed-strain aggregates and is required for competitor elimination within the *E. scolopes* light organ (13). Given that TasL facilitates contact in liquid environments, this protein may play a role in liquid supplemented with calcium.

Another possibility is that the aggregates observed here require a previously described polysaccharide. V. fischeri encodes two polysaccharide loci that promote biofilm formation: bacterial cellulose synthase (*bcs*) (25, 26) and symbiosis polysaccharide (*syp*) loci (27, 28). While little is known about the ecology of cellulose biofilm for V. fischeri, Syp biofilm is necessary for symbiosis establishment with juvenile *E. scolopes* squid (27, 28). Calcium was recently identified to promote both *bcs-*dependent biofilm in wild-type V. fischeri and *syp-*dependent biofilm in a *binK* mutant in liquid (17, 18). Therefore, it is conceivable that the aggregates observed here may have been due, at least in part, to biofilm.

### Conclusions.

Here, we have described evidence that calcium promotes T6SS competition in a low-viscosity liquid environment. Based on these and previous findings, we have expanded our existing model for conditional regulation of the V. fischeri T6SS2. Our data reveal that a combination of neutral pH and either low-viscosity liquid with seawater-like concentrations of calcium (10 mM) or host-like, high-viscosity liquid (15, 16) promotes T6SS expression and cell-cell contact in liquid to enable T6SS2 killing in a liquid environment (Fig. 2E). These findings show how bacteria sense and integrate multiple cues to evaluate the benefits of wielding a costly weapon and emphasize the importance of studying bacterial behaviors under ecologically relevant conditions. Furthermore, these data provide additional evidence that V. fischeri may use T6SS2 outside of the squid light organ (13).

### Methods.

See the supplemental material for a description of the methods used for this observation.
