# Supplementary material for: Calcium Promotes T6SS-Mediated Killing and Aggregation between Competing Symbionts
Source: Microbiol Spectr. 2022 Dec 1;10(6):e01397-22. doi: 10.1128/spectrum.01397-22 (PMC9769598; doi:10.1128/spectrum.01397-22)
Supplement: Supplemental file 1 — Supplemental material. Download spectrum.01397-22-s0001.pdf, PDF file, 0.2 MB [file spectrum.01397-22-s0001.pdf]

Supplemental Methods

Table S1. Strains and plasmids used in this study.

Strain	Relevant characteristics	Reference
ES114	*V. fischeri;* isolated from *Euprymna scolopes* light organ	Boettcher and Ruby 1994 (1)
ES401	*V. fischeri;* isolated from *E. scolopes* light organ	Lee 1994 (2)
ANS2100	ES401 with a disruption in *tssF_2* (Erm^R)	Speare *et al.*, 2020 (3)
**Plasmids**	**Relevant characteristics**	**Reference**
pVSV102	*gfp+*, $oriV_{R6k\gamma}$, $oriV_{pES213}$, *oriT*, Kn^R	Dunn *et al.*, 2006 (4)
pVSV208	*dsRed+*, $oriV_{R6k\gamma}$, $oriV_{pES213}$, *oriT*, Cm^R	“”
pSNS119	IPTG-inducible *vipA_2-gfp* fusion expression vector*;* $oriV_{R6K\gamma}$, $oriV_{pES213}$, *oriT*, Kn^R	Speare *et al.*, 2018 (5)

Media and growth conditions. *Vibrio fischeri* strains were grown in LBS liquid media at 24°C. Media was supplemented with 10mM $CaCl_2$, 10 mM NaCl, 10 mM $MgCl_2$ and/or 5% (w/v) polyvinylpyrrolidone (PVP360; Sigma-Aldrich) depending on the specific experiment. Antibiotic selection for *V. fischeri* strains are as follows: chloramphenicol (2 µg/ml), erythromycin (5 µg/ml), and kanamycin (100 µg/ml). Strains harboring pEVS102 and pEVS208 were grown in LBS supplemented with kanamycin and chloramphenicol, respectively.

Coincubation assays. Coincubation assays were performed as described previously (3). Briefly, overnight cultures of differentially-tagged strains were grown in LBS liquid supplemented with the appropriate antibiotic, shaking at 24°C or on LBS agar plates. Cell suspensions were then diluted to an OD_{600} of 1.0, mixed, and spotted into fresh LBS liquid media, and incubated shaking at 24°C. For experiments shown in Figure 1A, strains were mixed in a 1:1 ratio by volume and 10 µl of that mixture was added to 1 mL liquid media and incubated for 12 hours. For experiments shown in Figure 1B and 1C, strains were mixed in a 1:9 (ES114:ES401) ratio by volume and 30 µl of that mixture was added to 3 mL liquid media and incubated for 15 hours. Colony forming units (CFUs) for each strain were collected by plating serial dilutions of cocultures onto LBS plates with antibiotics to select for ES114 or ES401 at the beginning (T0) and end of each experiment and used to calculate log RCI (ES401/ES114) values (6).

Fluorescence Microscopy. Single-cell fluorescence microscopy was performed to visualize aggregates in LBS liquid, as described previously (6). Differentially-tagged strains were mixed in a 1:1 ratio for coculture aggregation assays. *V. fischeri* strains harboring either pVSV102 (GFP+) or pVSV208 (dsRed+) were incubated in LBS liquid + 10 mM $CaCl_2$. After 12 hours, 3 µl of each culture was spotted onto a glass slide and imaged with a 60 /1.3 numerical aperture oil Ph3 lens objective. Images were captured with an Olympus BX51 microscope outfitted with a Hamamatsu C8484-03G01 camera using MetaMorph software. Quantification of the ES401:ES114 ratio in either free-floating or aggregated cells was performed as described in Speare *et al.,* (7).

VipA_2-GFP sheath visualization was performed as described in Speare *et al* (5). Overnight cultures of cells harboring the IPTG-inducible *vipA_2-gfp* fusion expression vector (pSNS119) were added to LBS liquid (+/- 10 mM $CaCl_2$) supplemented with 0.5 mM IPTG and incubated for 3 hours. Cells were then spotted onto a glass slide and imaged as described above. Images were visualized and sheaths were quantified using Fiji software.

References

1. Boettcher KJ, Ruby EG. 1994. Occurrence of plasmid DNA in the sepiolid squid symbiont V*ibrio fischeri*. Current Microbiology 29:279-286.
2. Lee K-H, Ruby EG. 1994. Effect of the squid host on the abundance and distribution of symbiotic *Vibrio fischeri* in nature. Applied Environmental Microbiology 60:1565-1571.
3. Speare L, Smith S, Salvato F, Kleiner M, Septer AN. 2020. Environmental Viscosity Modulates Interbacterial Killing during Habitat Transition. mBio 11.
4. Dunn AK, Millikan DS, Adin DM, Bose JL, Stabb EV. 2006. New rfp-and pES213-derived tools for analyzing symbiotic *Vibrio fischeri* reveal patterns of infection and lux expression in situ. Applied Environmental Microbiology 72:802-810.
5. Speare L, Cecere AG, Guckes KR, Smith S, Wollenberg MS, Mandel MJ, Miyashiro T, Septer AN. 2018. Bacterial symbionts use a type VI secretion system to eliminate competitors in their natural host. Proc Natl Acad Sci U S A 115:E8528-E8537.
6. Speare L, Woo M, Bultman KM, Mandel MJ, Wollenberg MS, Septer AN. 2021. Host-Like Conditions Are Required for T6SS-Mediated Competition among Vibrio fischeri Light Organ Symbionts. Msphere 6:e01288-20.
7. Speare L, Woo M, Dunn AK, Septer AN. 2022. A Putative Lipoprotein Mediates Cell-Cell Contact for Type VI Secretion System-Dependent Killing of Specific Competitors. mBio:e03085-21.
